# Hypertension, blood pressure control and diabetic retinopathy in a large population-based study

**DOI:** 10.1371/journal.pone.0229665

**Published:** 2020-03-05

**Authors:** Lei Liu, Nguyen Duc Quang, Riswana Banu, Himeesh Kumar, Yih-Chung Tham, Ching-Yu Cheng, Tien Yin Wong, Charumathi Sabanayagam

**Affiliations:** 1 Singapore Eye Research Institute and Singapore National Eye Centre, Singapore, Singapore; 2 Department of Ophthalmology, The First Affiliated Hospital of China Medical University, Shenyang, China; 3 Alfred Health, Melbourne, Victoria, Australia; 4 Ophthalmology and Visual Sciences Academic Clinical Program, Duke-NUS Medical School, Singapore, Singapore; 5 Yong Loo Lin School of Medicine, National University of Singapore, Singapore, Singapore; Universidad Miguel Hernandez de Elche, SPAIN

## Abstract

**Background:**

Clinical trials have shown beneficial effects of blood pressure (BP) control in reducing the risk of diabetic retinopathy (DR). However, association between BP control and DR in population-based studies is not clear. We aimed to examine the association of hypertension and BP control with DR.

**Methods:**

We analysed data from a population-based cross-sectional study of Chinese, Malay and Indians adults with diabetes and hypertension (2004–2011, n = 2189, aged 40–80 years) in Singapore. DR severity was assessed from retinal photographs and graded for any- and vision-threatening DR (VTDR) using the modified Airlie House classification. Hypertension status was classified into (1) good control: on treatment (SBP < 130 and DBP < 80 mm Hg), (2) moderate control: on treatment, with BP levels other than group 1 and 3, (3) poor control: on treatment (SBP ≥140 and DBP ≥ 90 mm Hg), (4) untreated hypertension, any BP level. SBP, DBP and pulse pressure (PP) were analyzed as categories and as continuous variables. The association between BP and DR was assessed using multivariable logistic regression models.

**Results:**

The prevalence of any-DR and VTDR in the study population was 33.8% and 9.0% respectively. Both poorly controlled and untreated hypertension were significantly associated with any-DR with odds ratio (OR) (95% confidence interval [CI]) of 1.97 (1.39–2.83), and 2.01 [1.34–3.05]. Among BP components, SBP and PP were associated with both any-DR and VTDR with OR (95% CI) of 1.45 (1.28–1.65) and 1.61 (1.41–1.84) for any-DR, and 1.44 (1.19–1.76) and 1.67 (1.37–2.06) for VTDR.

**Conclusion:**

In a population-based sample of Asian adults with diabetes and hypertension, treated but poorly controlled as well as untreated hypertension were significantly associated with any-DR. Among the BP components, higher SBP and PP levels were associated with both any-DR and VTDR. Further longitudinal studies are necessary to confirm our findings.

## Introduction

Diabetic retinopathy (DR) is a common microvascular complication of diabetes. It is a major public health problem worldwide contributing to the majority of visual impairment among people with diabetes and a leading cause of blindness among working-age adults [1]. In 2010, one in three persons with diabetes was estimated to have some form of DR and one in 10 to have vision-threatening DR (VTDR) [2].

Apart from poor glycemic control, blood pressure (BP) has been shown to be an important risk factor for DR. Evidence from clinical trials has demonstrated beneficial effect of tight BP control on risk of DR in patients with diabetes and hypertension [3]. The 2017 Guideline for the Prevention, Detection, Evaluation, and Management of High Blood Pressure in Adults, recommend a BP goal of <130/80 mm Hg in adults with diabetes for prevention of further complications [4]. In population-based studies, while systolic blood pressure (SBP) has been consistently shown to be associated with DR [5–10], association of diastolic blood pressure (DBP) was less consistent with the majority showing no significant association of DBP with DR [5, 11], These studies examined BP or hypertension as one of the risk factors of DR, but none has examined in detail the association of hypertension treatment status, level of control or the range of SBP or DBP levels with DR. In addition, except for one population-based study, none has looked into the association of pulse pressure (PP, calculated as the difference between systolic and diastolic BP) with DR [12]. PP >60 mm Hg has been shown to be associated with increased risk of cardiovascular disease (CVD) and adverse renal outcomes in patients with type 2 diabetes [13, 14].

Ethnic differences have been reported in the prevalence and control of hypertension, as well as in DR prevalence. Of the three main ethnic groups in Singapore, Malays are reported to have higher prevalence of hypertension and poor BP control [15]. In addition, DR prevalence (~36%) was reported to be higher in Malay and Indian adults compared to Chinese with diabetes [16]. It is not clear if there are ethnic differences in the association between hypertension and DR. In this context, we aimed to examine the association of hypertension status, and BP components with presence and severity of DR in a multi-ethnic Asian population in Singapore.

## Methods

### Study population

Data for this study were derived from the Singapore Epidemiology of Eye Diseases (SEED), a population-based cross-sectional study of eye diseases in Chinese, Malay and Indian adults aged 40–80 years. Baseline data for Malays, Indians and Chinese were collected from 2004–2007, 2007–2009 and 2009–2011, respectively. Detailed methodology of SEED study has been published elsewhere [17, 18]. At baseline, 3,280 Malays, 3,400 Indians and 3,353 Chinese participated in the study with response rates of 78.7%, 75.6% and 72.8%, respectively. For this study, we included only participants with diabetes and hypertension (treated/untreated). Diabetes was defined as random glucose concentration ≥ 11.1 mmol/L, HbA_1_c ≥ 6.5%, self-reported anti-diabetic medication use, previously diagnosed and hypertension was defined as SBP ≥ 140 mm Hg or DBP ≥ 90 mm Hg or self-reported physician diagnosis of hypertension [19]. After excluding participants without fundus photography, data on BP, diabetes and other covariates included in the multivariate model, we included 2189 participants for the current analysis (Chinese, n = 459; Malays, n = 798; Indians, n = 932). This study was performed in accordance with the tenets of the Declaration of Helsinki and ethics approval was obtained from the Singapore Eye Research Institute Institutional Review Board. Written informed consent was provided by participants.

### Any-DR assessment

All participants underwent two-field color photographs (fields 1, centered on the optic disc; field 2, centered on the fovea) according to the Early Treatment for Diabetic Retinopathy Study (ETDRS) standard. Photographs were obtained from both eyes using a digital retinal camera (Canon CR-1 Mark-II Non-mydriatic Digital Retinal Camera; Canon, Tokyo, Japan) after pupil dilation with 1.0% tropicamide. DR severity was graded according to the modified Airlie House classification system [20]. Trained graders analysed the images for qualitative changes using a standardised protocol. Participants were classified as having retinopathy if their retinas had characteristic lesions defined by ETDRS severity scale: microaneurysms, haemorrhages, cotton wool spots, intra retinal microvascular abnormalities, hard exudates, venous beading, and new vessels. Based on the severity score of worse-eye, any-DR was defined as a score ≥ 15. DR severity was further classified as minimal non-proliferative (NPDR, level 15–20), mild NPDR (level 35), moderate NPDR (level 43–47), severe NPDR (level 53), and proliferative DR (PDR, score > 60) [21]. Vision-threatening DR (VTDR) was defined by the presence of severe NPDR, PDR, or clinically significant diabetic macular edema (DME).

### Assessment of hypertension

BP was measured with digital automatic BP monitor (Dinamap model Pro Series DP110X-RW, 100V2; GE Medical Systems Information Technologies, Inc., Milwaukee, WI) applied to the right arm of a seated participant after five-minute rest. Two BP measurements were taken 5 minutes apart, and if the two BP measurements differed by more than 10 mm systolic and 5 mm diastolic, a third measurement was taken and the average of two closest readings were taken as the BP value [22]. PP was calculated as the difference between SBP and DBP (SBP-DBP). Based on participants’ treatment and control status, hypertension was categorized into four groups (Group 1: good control on treatment with SBP <130 and DBP <80 mm Hg; Group 2: moderately controlled, on treatment with BP levels other than group 1 and 3; Group 3: poorly controlled, on treatment with SBP ≥140 and DBP ≥ 90 mm Hg, Group 4: untreated hypertension with any BP level). We kept ‘untreated hypertension’ as one category without specifying BP levels. It is possible that those with untreated hypertension may have BP within controlled levels via other means of control such as lifestyle modifications. Alternatively, it could also be due to newly diagnosed hypertension (abnormal BP levels detecting during the study visit). As the purpose of the study was to examine the association of hypertension treatment with DR, we did not stratify ‘untreated hypertension’ by BP levels.

### Assessment of covariates

Information on demographic, socioeconomic (education level and monthly household income), lifestyle (smoking and alcohol consumption) and medical history (diabetes, hypertension, and CVD) of the participants was collected during a comprehensive questionnaire-based interview. CVD was defined as self-reported physician diagnosed heart attack, angina, or stroke,. Body Mass Index (BMI) was calculated as weight in kilograms (kg) divided by height in meters (m) squared. A 40-ml sample of non-fasting venous blood was collected to determine glycated haemoglobin (HbA_1_c), random glucose, total cholesterol and high density lipoprotein (HDL) cholesterol concentrations. Chronic kidney disease (CKD) was defined as an estimated glomerular filtration rate (eGFR) < 60 mL/min/1.73 m^2^ [23]. eGFR was calculated from the serum creatinine levels according to the CKD Epidemiology Collaboration (CKD-EPI) equation [24].

### Statistical analysis

We used R software version 3.4.2 (R system for statistical computing, available from Comprehensive R Archive Network http://cran.r-project.org/) for our statistical analyses. BP components (SBP, DBP and PP) were analyzed as both continuous (per SD increase) and categorical variables (quartiles). Hypertension status was examined in all four categories as defined above. For outcome VTDR, due to the smaller number of events in-groups 3 (n = 11) and 4 (n = 57), we combined groups 2, 3 and 4 as uncontrolled hypertension. Associations of hypertension status and BP components (SBP, DBP and PP) with any-DR were analysed using two logistic regression models: model 1, adjusted for age, sex, ethnicity; model 2 with additional adjustment for socioeconomic (education, income level), lifestyle (current smoking status, alcohol consumption, BMI), and clinical factors (total and HDL cholesterol, duration of diabetes, anti-diabetic medication use, HbA_1_c, anti-hypertension medication use, CVD and CKD status). Both regression models were also used to analyse the associations between hypertension/BP components and VTDR. To examine the consistency of the associations, we performed subgroup analysis stratified by ethnicity. Statistical interaction between hypertension categories and the stratifying variable (ethnicity) was examined in the corresponding logistic regression model by including cross-product interaction terms. To examine the dose-response relationship between SBP, PP level and any-DR, and VTDR without linearity assumptions, we used generalised additive modelling approach (flexible nonparametric logistic regression) to calculate odds of any-DR and VTDR, adjusting for all covariates in the multivariate model. Predicted odds of any-DR and VTDR were then plotted against increasing SBP and PP levels (on log scale).

## Results

**Fig 1:**
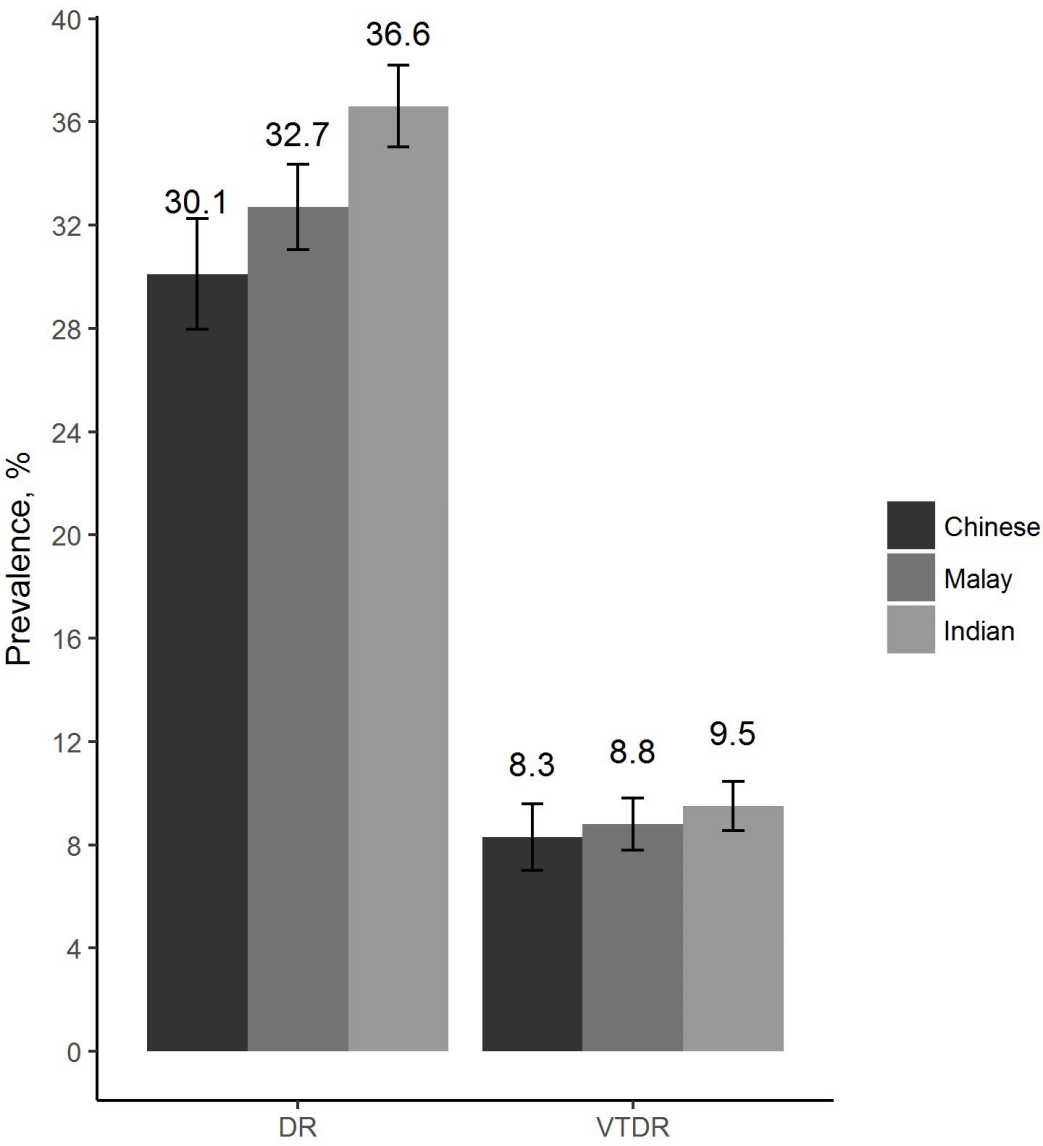
Prevalence of any-DR and VTDR stratified by ethnicity.

The prevalence of any-DR and VTDR in our study population was 33.8% (n = 740) and 9.0% (n = 197), respectively. As shown in **Fig 1**, no significant differences were observed in the crude prevalence of any-DR and VTDR in the three ethnic groups (*P* = 0.2 for any-DR, *P* = 0.8 for VTDR). **Table 1** summarised the baseline characteristics of participants stratified by hypertension categories. Of the 2,189 participants with hypertension, 279 (12.8%) had controlled hypertension, 1,046 (47.8%) had moderately controlled hypertension, 139 (6.4%) had poorly controlled hypertension, and 725 (33.1%) had untreated hypertension. Participants with treated and controlled hypertension were less likely to be Malays, primary/below educated and to have monthly income <S$1,000, had higher prevalence of CVD, anti-diabetic medication use, and had lower levels of SBP, DBP, PP, HbA_1_c, total and HDL cholesterol. Those with treated, but moderately controlled hypertension were older, less likely to be currently smoking, and more likely to have monthly income <S$1,000. Those on treatment and with poorly controlled hypertension had higher prevalence of CKD, higher levels of all three BP components (SBP, DBP, PP) and HDL cholesterol. Those with untreated naïve hypertension were younger, mostly Malays, current smokers, primary/below educated, had lower prevalence of CVD, CKD and anti-diabetic medication use, had higher levels of HbA_1_c, and total cholesterol. There were no significant differences in sex, alcohol consumption, diabetes duration, and BMI levels across the four hypertension categories.

**Table 1:** Baseline characteristics of participants stratified by hypertensive groups.

Variables	Overall (n = 2189)	Treated, controlled (n = 279)	Treated, moderately controlled (n = 1046)	Treated, poorly controlled (n = 139)	Untreated (n = 725)	P*
Age, years	60.6 (9.9)	62.0 (9.9)	64.5 (9.0)	60.8 (7.8)	60.63 (9.9)	<0.001
Male, %	1104 (50.4)	153 (54.8)	499 (47.7)	74 (53.2)	378 (52.1)	0.09
Ethnicity, %						
Chinese	459 (21.0)	72 (15.7)	260 (56.6)	17 (3.7)	110 (24.0)	<0.001
Malay	798 (36.4)	46 (5.8)	333 (41.7)	65 (8.1)	354 (44.4)	
Indian	932 (42.6)	161 (20.2)	453 (56.8)	57 (7.1)	261 (32.7)	
Primary or below education, %	1567 (71.6)	178 (63.8)	740 (70.7)	102 (73.4)	547 (75.4)	0.003
Monthly income, <S$1,000, %	1425 (65.1)	163 (58.4)	719 (68.7)	87 (62.6)	456 (62.9)	0.004
Current smoking, %	267 (12.2)	30 (10.8)	88 (8.4)	16 (11.5)	133 (18.3)	<0.001
Alcohol consumption, %	169 (7.7)	25 (9)	71 (6.8)	11 (7.9)	62 (8.6)	0.5
History of CVD, %	473 (21.6)	103 (36.9)	264 (25.2)	27 (19.4)	79 (10.9)	<0.001
CKD, %	489 (22.3)	60 (21.5)	276 (26.4)	38 (27.3)	115 (15.9)	<0.001
Use of anti-diabetic medication, %	1389 (63.5)	216 (77.4)	791 (75.6)	99 (71.2)	283 (39)	<0.001
Diabetes duration, years	10.2 (9.3)	10.5 (9.0)	11.5 (9.2)	8.9 (8.1)	10.2 (9.3)	0.08
SBP, mm Hg	157.4 (19.4)	118.7 (8.4)	149.9 (15.6)	171.0 (18.0)	157.4 (19.4)	<0.001
DBP, mm Hg	83.6 (11.2)	68.2 (5.5)	76.6 (7.3)	96.7 (7.2)	83.6 (11.2)	<0.001
PP, mm Hg	73.8 (16.7)	50.5 (8.5)	73.3 (15.7)	74.2 (16.2)	73.8 (16.7)	<0.001
HbA_1_c, %	8.2 (2)	7.2 (1.2)	7.5 (1.5)	7.7 (1.5)	8.2 (2.0)	<0.001
BMI, kg/m^2^	26.7 (4.7)	27.0 (4.7)	27.2 (4.9)	27.5 (4.4)	26.8 (4.7)	0.1
HDL-cholesterol, mmol/L	1.2 (0.3)	1.1 (0.3)	1.2 (0.3)	1.2 (0.3)	1.2 (0.3)	<0.001
Total cholesterol, mmol/L	5.7 (1.3)	4.4 (1.0)	4.9 (1.1)	5.4 (1.3)	5.7 (1.3)	<0.001

Abbreviations: BMI, body mass index; CKD: chronic kidney disease; CVD, cardiovascular disease; DBP: diastolic blood pressure; HDL: high-density lipoprotein; PP, pulse pressure; SBP: systolic blood pressure; SD, standard deviation

*P-values for continuous variables were obtained using ANOVA, and for categorical variables using chi-square tests.

Data presented are frequency (percentage) or mean (standard deviation) as appropriate for the variable.

### Association between hypertension, BP components and any-DR

**Fig 2:**
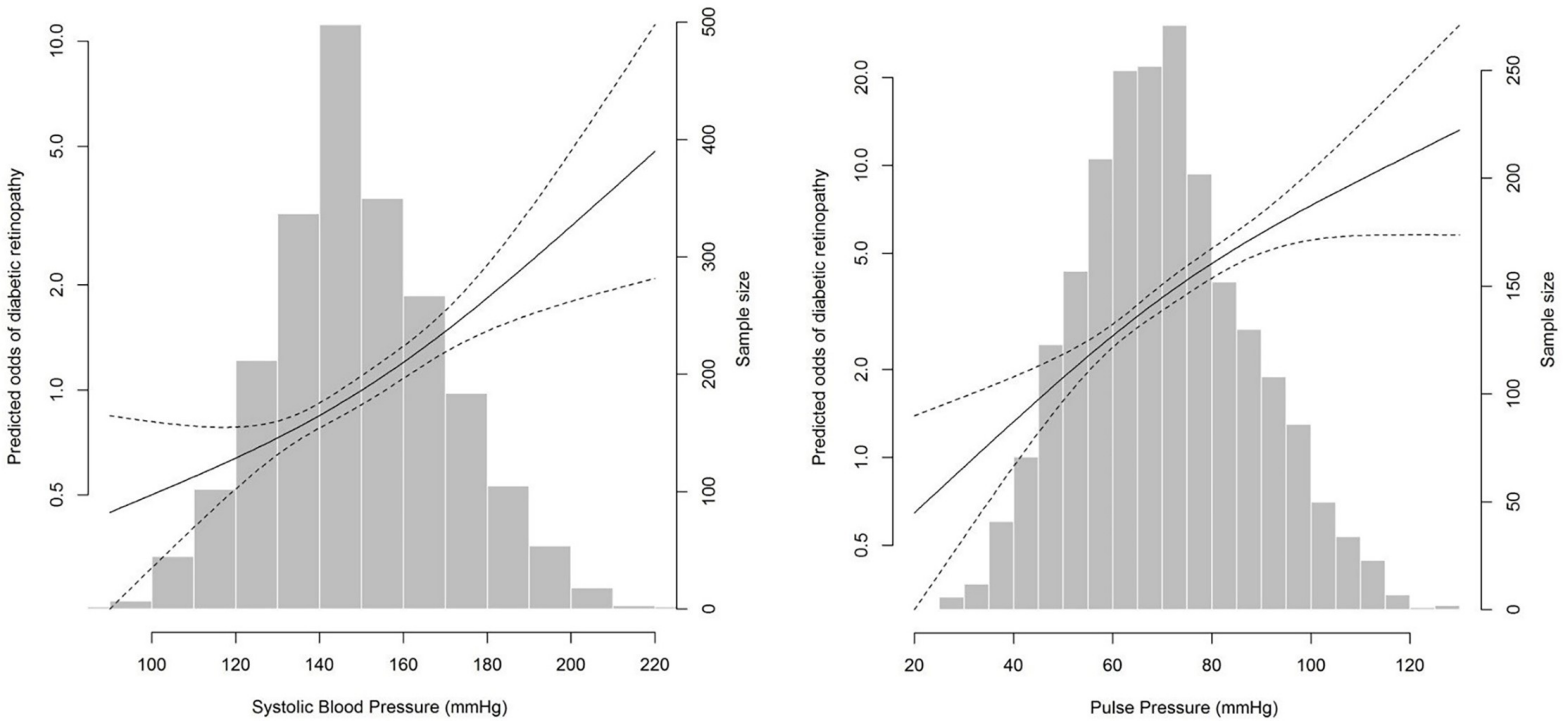
Associations between systolic blood pressure (SBP), pulse pressure (PP) and any diabetic retinopathy (DR). Multivariate-adjusted odds of any-DR according to: **(A)** SBP and **(B)** PP. Black line represents predicted odds of any-DR from non-parametric logistic regression. Dashed lines represent 95% confidence limits for the nonparametric logistic regression estimates. The nonparametric logistic regression was adjusted for age, gender, ethnicity, diabetic duration, anti-diabetic medication use, total cholesterol, HDL-cholesterol, HbA_1_c, current smoking status, current alcoholic consumption, education level, income level, anti-hypertension medication use, BMI, history of CVD and CKD status.

**Table 2** shows the association between BP categories and any-DR. After adjusting for age, sex and ethnicity (model 1), only treated and poorly controlled hypertension was associated with any-DR. After adjusting for potential confounders (model 2), both treated and poorly controlled (odds ratio [OR] = 1.97), and untreated hypertension (OR = 2.01) were associated with any-DR. Quartile analysis of individual BP components showed that any-DR prevalence increased with increasing levels of both SBP and PP (*P*< 0.001). Both categorical and continuous logistic regression analyses showed that SBP and PP were associated with any-DR in both regression models. Association between SBP and PP levels and any-DR remained positive when SBP and PP were analysed as continuous variables. However, there was no significant association between DBP (categorical as well as continuous) and any-DR in either model. In nonparametric models without linearity assumptions, continuous positive associations were observed between SBP (**Fig 2A**) and PP (**Fig 2B**) levels with any-DR.

**Table 2:** Association between hypertension, BP categories and DR.

BP categories	Number at risk (cases)	DR, %	Age, sex, and ethnicity-adjusted OR (95% CI)	Multivariate* OR (95% CI)
**Hypertension categories**				
Treated, controlled	279 (77)	27.6	Ref	Ref
Treated, moderately controlled	1046 (379)	36.2	0.93 (0.64–1.34)	0.97 (0.63–1.50)
Treated, poorly controlled	139 (59)	42.5	1.87 (1.39–2.54)	1.97 (1.39–2.83)
Untreated	725 (225)	31.0	1.24 (0.91–1.70)	2.01 (1.34–3.05)
**SBP Quartile**				
Quantile 1 (84.5–136 mm Hg)	559 (154)	27.6	Ref	Ref
Quantile 2 (136.5–148 mm Hg)	536 (163)	30.4	1.19 (0.92–1.56)	1.37 (0.98–1.91)
Quantile 3 (148.33–163 mm Hg)	553 (191)	34.5	1.50 (1.15–1.95)	1.89 (1.35–2.65)
Quantile 4 (163.5–275 mm Hg)	540 (231)	42.8	2.20 (1.67–2.91)	2.60 (1.78–3.80)
*P* trend		<0.001	<0.001	<0.001
Each SD increase in SBP			1.38 (1.26–1.52)	1.45 (1.28–1.65)
**DBP Quartile**				
Quantile 1 (43–70.5 mm Hg)	560 (199)	35.5	Ref	Ref
Quantile 2 (70.67–78 mm Hg)	537 (193)	35.9	1.00 (0.78–1.29)	1.26 (0.92–1.74)
Quantile 3 (78.33–86 mm Hg)	559 (178)	31.8	0.87 (0.67–1.13)	1.00 (0.71–1.39)
Quantile 4 (86.5–131 mm Hg)	532 (169)	31.8	0.94 (0.71–1.24)	1.24 (0.83–1.87)
*P* trend		0.09	0.2	0.8
Each SD increase in DBP			1.0 (0.86–1.05)	1.04 (0.91–1.19)
** PP Quartile**				
Quartile 1 (25–58.5 mm Hg)	563 (134)	23.8	Ref	Ref
Quartile 2 (58.67–70 mm Hg)	535 (148)	27.7	1.32 (1.00–1.76)	1.53 (1.07–2.18)
Quartile 3 (70.17–82 mm Hg)	550 (219)	39.8	2.28 (1.72–3.03)	2.91 (2.03–4.2)
Quartile 4 (82.5–144 mm Hg)	540 (238)	44.1	3.16 (2.31–4.35)	3.43 (2.27–5.23)
*P* trend		< 0.001	<0.001	<0.001
Each SD increase in PP			1.64 (1.48–1.82)	1.61 (1.41–1.84)

Abbreviations: BP, blood pressure; CI, confidence interval; DBP, diastolic blood pressure; DR, diabetic retinopathy; OR, odds ratio; PP, pulse pressure; SBP, systolic blood pressure; SD, standard deviation.

*Adjusted for age, gender, ethnicity, education level, income level, current smoking, current alcohol consumption, body mass index, total cholesterol, HDL cholesterol, HbA_1_c, chronic kidney disease, history of cardiovascular disease, duration of diabetes, anti-diabetic medication use, and anti-hypertensive medication use.

*P*-interaction between hypertension categories and ethnicity in multivariate model = 0.009; SBP and ethnicity in multivariate model = 0.1; PP and ethnicity in multivariate model = 0.6.

### Association between hypertension, BP components and VTDR

**Fig 3:**
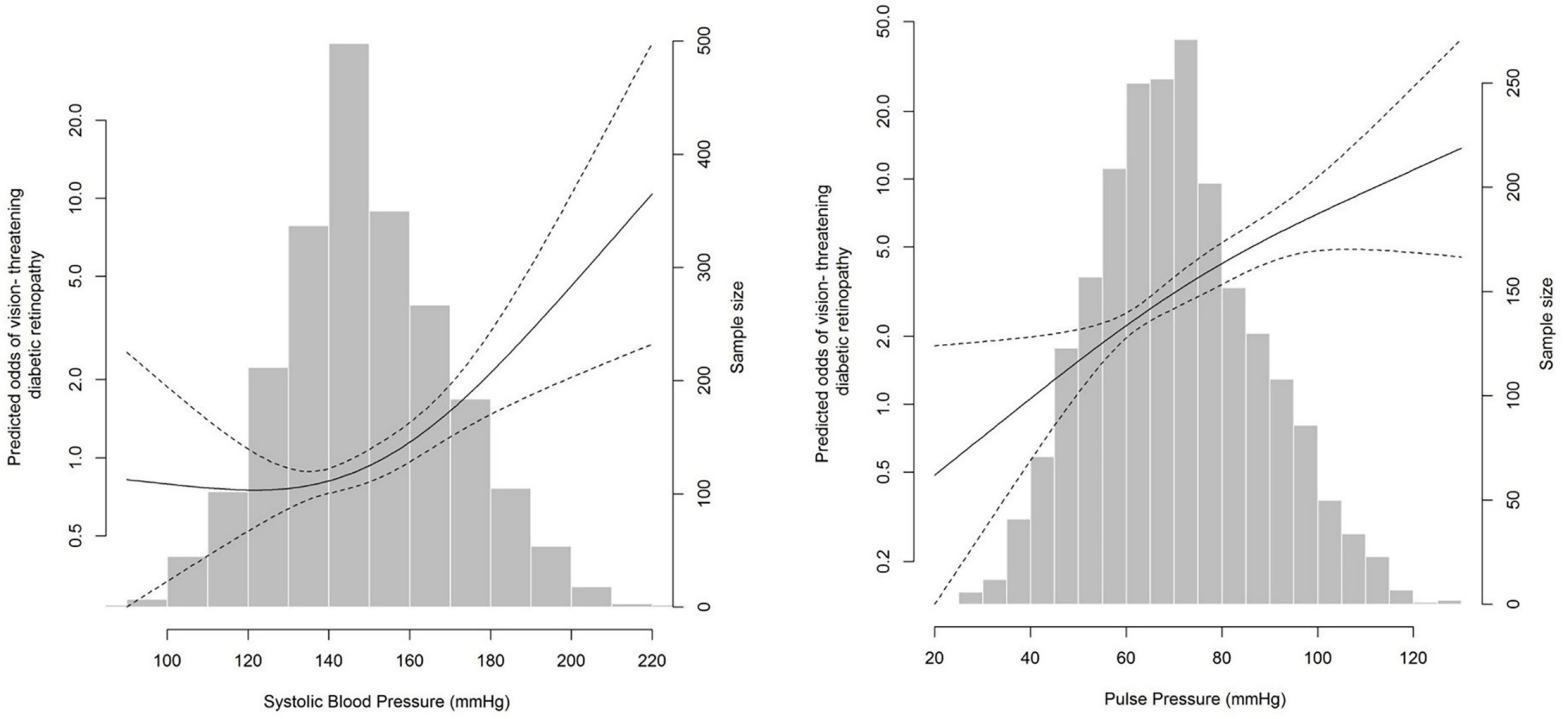
Associations between systolic blood pressure (SBP), pulse pressure (PP) and vision-threatening diabetic retinopathy (VTDR). Multivariate -adjusted odds of VTDR according to: **(A)** SBP and **(B)** PP. Black line represents the predicted odds of any-DR from non-parametric logistic regression. Dashed lines represent 95% confidence limits for the nonparametric logistic regression estimates. The nonparametric logistic regression was adjusted for age, gender, ethnicity, diabetic duration, anti-diabetic medication use, total cholesterol, HDL-cholesterol, HbA_1_c, current smoking status, current alcoholic consumption, education level, income level, anti-hypertension medication use, BMI, history of CVD and CKD status.

Compared to the reference (treated and controlled), untreated/uncontrolled hypertension was not associated with VTDR in either multivariate models (**Table 3**). Among the BP components, increasing levels of both SBP and PP levels were significantly associated with VTDR in both models. In non-parametric models, SBP levels higher than 140 mm Hg (**Fig 3A**) and PP levels higher than 75 mm Hg (**Fig 3B**) were significantly associated with VTDR.

**Table 3:** Association between BP categories and VTDR.

BP categories	Number at risk (cases)	DR, %	Age, sex, and ethnicity-adjusted OR (95% CI)	Multivariate* OR (95% CI)
**Hypertension categories**				
Treated, controlled	279 (22)	7.9	Ref	Ref
Treated, uncontrolled /untreated	1910 (175)	9.2	1.18 (0.75–1.93)	1.30 (0.75–2.35)
**SBP Quartile**				
Quantile 1 (84.5–136 mm Hg)	559 (39)	7.0	Ref	Ref
Quantile 2 (136.5–148 mm Hg)	536 (29)	5.4	0.78 (0.47–1.28)	1.18 (0.63–2.18)
Quantile 3 (148.33–163 mm Hg)	553 (49)	8.9	1.47 (0.94–2.31)	2.06 (1.17–3.66)
Quantile 4 (163.5–275 mm Hg)	540 (80)	14.8	2.41 (1.56–3.76)	2.90 (1.59–5.39)
*P* trend		< 0.001	< 0.001	< 0.001
Each SD increase in SBP			1.56 (1.34–1.81)	1.44 (1.19–1.76)
**DBP Quartile**				
Quantile 1 (43–70.5 mm Hg)	560 (11.8)	11.8	Ref	Ref
Quantile 2 (70.67–78 mm Hg)	537 (7.5)	7.5	0.57 (0.37–0.86)	0.80 (0.47–1.34)
Quantile 3 (78.33–86 mm Hg)	559 (8.6)	8.6	0.65 (0.43–0.98)	0.98 (0.59–1.65)
Quantile 4 (86.5–131 mm Hg)	532 (8.1)	8.1	0.66 (0.42–1.03)	0.94 (0.47–1.87)
*P* trend		0.06	0.08	0.7
Each SD increase in DBP			0.88 (0.75–1.04)	0.94 (0.75–1.15)
**PP Quartile**				
Quantile 1 (25–58.5 mm Hg)	563 (26)	4.6	Ref	Ref
Quantile 2 (58.67–69.5 mm Hg)	535 (27)	5.1	1.25 (0.70–2.23)	1.56 (0.78–3.16)
Quantile 3 (69.83–81.5 mm Hg)	550 (60)	10.9	2.89 (1.75–4.89)	3.62 (1.96–6.91)
Quantile 4 (82–144 mm Hg)	540 (84)	15.6	4.32 (2.56–7.50)	3.99 (2.04–8.06)
*P* trend		< 0.001	< 0.001	< 0.001
Each SD increase in PP			2.02 (1.71–2.39)	1.67 (1.37–2.06)

Abbreviations: BP, blood pressure; CI, confidence interval; DBP, diastolic blood pressure; DR, diabetic retinopathy; OR, odds ratio; PP, pulse pressure; SBP, systolic blood pressure; VTDR, vision-threatening DR.

*Adjusted for age, gender, ethnicity, education level, income level, current smoking, current alcohol consumption, body mass index, total cholesterol, HDL cholesterol, HbA_1_c, chronic kidney disease, history of cardiovascular disease, duration of diabetes, anti-diabetic medication use, and anti-hypertensive medication use.

*P*-interaction between hypertension categories and ethnicity in multivariate model = 0.691; SBP and ethnicity in multivariate model = 0.1; DBP and ethnicity in multivariate model = 0.3.

### Association between hypertension categories and any-DR by ethnicity

There was significant interaction by ethnicity in the association between hypertension categories and any-DR (*P*-interaction = 0.009, **Table 4**). While, both treated and poorly controlled (OR: 4.3, 95% confidence interval [CI]:1.62–13.70), and untreated hypertension (OR: 4.76, 95% [CI]:1.72–15.66) were significantly associated with any-DR in Malays, treated and poorly controlled (OR: 2.15, 95% [CI]:1.31–3.56) showed significant association in Indians and none of the categories were significant in Chinese.

**Table 4:** Association between hypertension categories and DR stratified by ethnic groups.

Hypertension categories	Number at risk (cases)	DR, %	Age, sex, and ethnicity-adjusted OR (95% CI)	Multivariate* OR (95% CI)
**Chinese**				
Treated, controlled	72 (23)	31.9	Ref	Ref
Treated, moderately controlled	260 (73)	28.1	0.35 (0.16–0.76)	0.31 (0.12–0.77)
Treated, poorly controlled	17 (6)	35.3	1.20 (0.67–2.18)	1.36 (0.67–2.79)
Untreated	110 (36)	32.7	1.01 (0.53–1.92)	1.61 (0.69–3.79)
**Malay**				
Treated, controlled	46 (8)	17.4	Ref	Ref
Treated, moderately controlled	333 (118)	35.4	1.36 (0.52–3.72)	1.85 (0.55–6.91)
Treated, poorly controlled	65 (27)	41.5	2.96 (1.40–7.03)	4.30 (1.62–13.70)
Untreated	354 (108)	30.5	2.15 (1.02–5.12)	4.76 (1.72–15.66)
**Indian**				
Treated, controlled	161 (46)	28.6	Ref	Ref
Treated, moderately controlled	453 (188)	41.5	1.28 (0.79–2.06)	1.31 (0.73–2.34)
Treated, poorly controlled	57 (26)	45.6	2.09 (1.4–3.14)	2.15 (1.31–3.56)
Untreated	261 (81)	31.0	1.13 (0.74–1.75)	1.67 (0.92–3.07)

Abbreviations: CI, confidence interval; DBP, diastolic blood pressure; DR, diabetic retinopathy; OR, odds ratio; PP, pulse pressure; SBP, systolic blood pressure; SD, standard deviation

*Adjusted for age, gender, education level, income level, current smoking, current alcohol consumption, body mass index, total cholesterol, HDL cholesterol, HbA_1_c, chronic kidney disease, history of cardiovascular disease, duration of diabetes, anti-diabetic medication use, and anti-hypertensive medication use.

### Association between BP components and any-DR, and VTDR by ethnicity

**Table 5** shows association between BP components and any-DR and VTDR stratified by ethnicity. Similar to the main analysis, both SBP and PP levels were significantly associated with any-DR in all three ethnic groups. With regards to VTDR, SBP and PP levels showed significant association in Chinese and Malays. In Indians, neither SBP nor PP showed significant association with VTDR. However, there was no significant interaction by ethnicity in the association of SBP (*P*-interaction = 0.1), or PP (*P*-interaction = 0.6) with any-DR or VTDR.

**Table 5:** Association between BP components and DR/VTDR stratified by ethnicity.

	Number at risk (cases)	Cases, %	Multivariate OR* (95% CI) (per SD increase)		
			SBP	DBP	PP
DR					
Chinese	459 (138)	30.1	1.87 (1.35–2.61)	1.15 (0.80–1.64)	2.06 (1.48–2.93)
Malay	797 (261)	32.7	1.57 (1.27–1.95)	1.06 (0.85–1.32)	1.85 (1.46–2.37)
Indian	932 (341)	36.6	1.29 (1.06–1.57)	1.01 (0.82–1.23)	1.38 (1.13–1.70)
VTDR					
Chinese	459 (38)	8.3	1.73 (1.01–2.98)	1.03 (0.54–1.89)	1.89 (1.11–3.26)
Malay	798 (70)	8.8	1.83 (1.33–2.57)	0.94 (0.67–1.31)	2.46 (1.71–3.63)
Indian	932 (89)	9.6	1.10 (0.80–1.51)	0.90 (0.64–1.26)	1.20 (0.87–1.65)

Abbreviations: BP, blood pressure; CI, confidence interval; DBP, diastolic blood pressure; DR, diabetic retinopathy; OR, odds ratio; PP, pulse pressure; SBP, systolic blood pressure; VTDR, vision-threatening DR

*Adjusted for age, gender, education level, income level, current smoking, current alcohol consumption, body mass index, total cholesterol, HDL cholesterol, HbA_1_c, chronic kidney disease, history of cardiovascular disease, duration of diabetes, anti-diabetic medication use, and anti-hypertensive medication use.

## Discussion

In this population-based sample of Chinese, Malay and Indian adults with diabetes and hypertension both treated but poorly controlled and untreated hypertension were significantly associated with any-DR compared to treated and good control (SBP <130 and DBP <80 mm Hg). After adjusting for potential confounders, elevated levels of SBP and PP were associated with both any-DR and VTDR. In non-parametric models, for any-DR, while a continuous positive association was observed across the SBP and PP levels, the linear association for VTDR was evident for participants with SBP levels above 140 mm Hg and PP above 75 mm Hg. In subgroup analyses stratified by ethnicity, hypertension categories were significant in Malay and Indians but not in Chinese, but associations of SBP and PP with any-DR were consistently present in all three ethnic groups.

To the best of our knowledge, our study is the first to examine the association of hypertension treatment with DR using population-based samples. Several clinical trials have examined the effect of hypertension control on the incidence and progression of DR. While some have reported beneficial effect of intensive control on DR risk, others have shown no significant effect. It must be noted that the definition of BP control is not standard across the BP studies and this might have influenced the conclusions about the effect of BP control on retinopathy. For instance, in the UK Prospective Diabetes Study (UKPDS), tight BP control (SBP below 143 mm Hg) in patients with type 2 diabetes was beneficial in preventing any-DR progression [3]. Similarly, a recent retrospective cohort study in Taiwanese population with type 2 diabetes revealed that pre-morbid hypertension and poor SBP control were associated with development of new DR [25]. In contrast, the ADVANCE study, a randomised controlled trial, showed that BP control within the normal range (below 140/80 mm Hg) had no effect in preventing DR progression [26]. Furthermore, the Appropriate Blood Pressure Control in Diabetes (ABCD) trial reported no difference between intensive (DBP goal of 75 mm Hg) and moderate (DBP goal of 80–89 mm Hg) BP control with DR progression in hypertensive type 2 diabetic subjects [27, 28]. Longitudinal studies involving hypertensive patients from populations are necessary to confirm our findings.

We observed significant ethnic differences between hypertension categories and any-DR (p-interaction by ethnicity in the association between hypertension categories and any-DR = 0.009) in our study. While there was an association between poorly controlled hypertension and any-DR in both Malays (multivariable OR of 4.30 [1.62–13.70]) and Indians (2.15 [1.31–3.56]), this was not observed in the Chinese population (1.36 [0.67–2.79]). In the current study, among those with hypertension on treatment, Chinese hypertensives were found to have lowest proportion of poorly controlled hypertension (4.9% in Chinese vs. 14.6% in Malays and 8.5% in Indians). This is consistent with previous report from the Singapore National Health Survey 2010 [29] which showed that among those with known hypertension, Chinese had the highest proportion of good control (70%) followed closely by Indians (68.9%) compared to Malays (51.5%). In addition, since prevalence of diabetes was significantly lower in Chinese (15.2%) compared to Malays (24.4%) and Indians (34.5%) in the SEED study [30], number of Chinese participants (n = 459) included for the current analysis was also relatively small compared to Malays (n = 798) and Indians (n = 932). Consequently, only 17 Chinese participants fall under ‘treated, poorly controlled’ category of which only 6 had DR. Lower proportion of those with poorly controlled hypertension and the small number of DR events due to lower prevalence of diabetes in Chinese could possibly explain the lack of association between hypertension category and any-DR in Chinese.

In the current study, higher SBP levels were associated with both any-DR and VTDR. Our findings on SBP and any-DR are similar to reports from several cross-sectional studies conducted in India, and China [7, 31–34]. A cross-sectional study conducted in India involving rural population showed similar significant associations between SBP and presence and severity of any-DR [7]. Another cross-sectional study involving urban participants in India, showed increasing SBP to be associated with any-DR in newly diagnosed type 2 diabetes patients [33]. Similarly, higher SBP was also an independent risk factor for both any-DR and VTDR in Indians who migrated to an urbanised country such as Singapore [34]. In two cross-sectional studies in China, higher SBP was also an independent risk factor for both any-DR [31, 32] and sight-threatening DR [32].

Similar to SBP, we found higher PP levels to be associated with both any-DR and VTDR. PP levels were shown to be associated with any-DR in both UKPDS [35] and a Congo based study [36]. The outcomes were consistent with the findings in our study in which PP was associated with VTDR. Abnormal PP has been reported to increase blood flow shear stress and destroy the retinal capillary endothelial cells in diabetic eyes [37], which may explain the association between PP and VTDR. Elevated PP levels have also been associated with micro- and macrovascular complications in patients with type 2 diabetes [38]. Compared to SBP, PP has been demonstrated as a better predictor of coronary heart disease (CHD) in persons with type 2 diabetes [13]. In hypertensive patients, PP may be used as a marker of preclinical cardiovascular disease such as pre-clinical atherosclerosis and arterial stiffness [39].

We found that DBP level was not associated with any-DR and VTDR. Unlike SBP, DBP with any-DR association has not been consistently observed in the literature. Similar to our findings, two cross-sectional studies conducted in China reported no significant associations between DBP and any-DR [31, 40]. However, a previous study using four fields of digital retinal colour photography to detect any-DR in Asian Indians showed DBP to be associated with any-DR in type 2 diabetes [41]. In the Wisconsin Epidemiologic Study of Diabetic Retinopathy (WESDR), no significant association was found between DBP and DR progression in type 2 diabetic patients [42]. It is possible that antihypertensive treatment would have reduced diastolic BP more than systolic BP. For e.g. in patients with chronic kidney disease and hypertension, it has been shown that in hypertensive patients, treatment with multiple antihypertensive drugs was associated with lowering of diastolic BP more than systolic BP [43].

Strengths of this study include the use of large, population-based, multi-ethnic sample of diabetic participants with hypertension. However, there are limitations that might affect the interpretation of the outcomes. First, this is a cross-sectional study that limits inference of causality or temporality in the associated outcomes. Second, although we had adjusted for anti-hypertensive medication use, we did not collect information on the dosage, class and duration of use. Third, we used BP measurements from a single visit and this could have over-estimated hypertension prevalence. Lastly, in diabetic participants, diabetic glomerulopathy leads to difficult control of hypertension and diabetic glomerular disease might explain the poor BP control among those with renal insufficiency. Unfortunately, information on albuminuria was missing in nearly a third of the participants, thus, we could not account for the effect of diabetic glomerulopathy in poor BP control. However, adjustment for eGFR, a kidney function marker, in multivariable models suggest that the observed association between poor BP control and DR is independent of eGFR and other conventional risk factors including antihypertensive medication use which prevents progression of albuminuria.

In conclusion, in a multi-ethnic sample of Asian adults with diabetes and hypertension, both treated and poorly controlled and untreated hypertension were associated with any-DR. Higher levels of SBP and PP were associated with any-DR and VTDR. Our findings suggest that in participants with both diabetes and hypertension, a tighter control of BP may help prevent DR.

## References

[1] Bunce C, Wormald R. Causes of blind certifications in England and Wales: April 1999-March 2000. Eye (Lond). 2008;22(7):905–11. 10.1038/sj.eye.6702767 . 17332762

[2] Yau JW, Rogers SL, Kawasaki R, Lamoureux EL, Kowalski JW, Bek T, et al Global prevalence and major risk factors of diabetic retinopathy. Diabetes Care. 2012;35(3):556–64. 10.2337/dc11-1909 22301125 PMC3322721

[3] Tight blood pressure control and risk of macrovascular and microvascular complications in type 2 diabetes: UKPDS 38. UK Prospective Diabetes Study Group. BMJ. 1998;317(7160):703–13. 9732337 PMC28659

[4] Whelton PK, Carey RM, Aronow WS, Casey DE Jr., Collins KJ, Dennison Himmelfarb C, et al 2017 ACC/AHA/AAPA/ABC/ACPM/AGS/APhA/ASH/ASPC/NMA/PCNA Guideline for the Prevention, Detection, Evaluation, and Management of High Blood Pressure in Adults: A Report of the American College of Cardiology/American Heart Association Task Force on Clinical Practice Guidelines. J Am Coll Cardiol. 2017. Epub 2017/11/18. 10.1016/j.jacc.2017.11.006 . 29146535

[5] Klein R, Sharrett AR, Klein BE, Moss SE, Folsom AR, Wong TY, et al The association of atherosclerosis, vascular risk factors, and retinopathy in adults with diabetes: the atherosclerosis risk in communities study. Ophthalmology. 2002;109(7):1225–34. 10.1016/s0161-6420(02)01074-6 . 12093643

[6] Kramer CK, Leitao CB, Canani LH, Ricardo ED, Pinto LC, Valiatti FB, et al Late afternoon blood pressure increase is associated with diabetic retinopathy in normotensive type 2 diabetes mellitus patients. Diabetes Res Clin Pract. 2009;84(1):e12–4. Epub 2009/02/03. 10.1016/j.diabres.2008.12.016 . 19181416

[7] Rani PK, Raman R, Chandrakantan A, Pal SS, Perumal GM, Sharma T. Risk factors for diabetic retinopathy in self-reported rural population with diabetes. J Postgrad Med. 2009;55(2):92–6. 10.4103/0022-3859.48787 . 19550052

[8] Sasongko MB, Wong TY, Nguyen TT, Shaw JE, Jenkins AJ, Wang JJ. Novel versus traditional risk markers for diabetic retinopathy. Diabetologia. 2012;55(3):666–70. 10.1007/s00125-011-2424-x . 22198262

[9] Wang FH, Liang YB, Peng XY, Wang JJ, Zhang F, Wei WB, et al Risk factors for diabetic retinopathy in a rural Chinese population with type 2 diabetes: the Handan Eye Study. Acta Ophthalmol. 2011;89(4):e336–43. 10.1111/j.1755-3768.2010.02062.x . 21371287

[10] Wong TY, Klein R, Islam FM, Cotch MF, Folsom AR, Klein BE, et al Diabetic retinopathy in a multi-ethnic cohort in the United States. Am J Ophthalmol. 2006;141(3):446–55. 10.1016/j.ajo.2005.08.063 16490489 PMC2246042

[11] van Leiden HA, Dekker JM, Moll AC, Nijpels G, Heine RJ, Bouter LM, et al Blood pressure, lipids, and obesity are associated with retinopathy: the hoorn study. Diabetes Care. 2002;25(8):1320–5. 10.2337/diacare.25.8.1320 . 12145228

[12] Wong TY, Cheung N, Tay WT, Wang JJ, Aung T, Saw SM, et al Prevalence and risk factors for diabetic retinopathy: the Singapore Malay Eye Study. Ophthalmology. 2008;115(11):1869–75. 10.1016/j.ophtha.2008.05.014 . 18584872

[13] Cockcroft JR, Wilkinson IB, Evans M, McEwan P, Peters JR, Davies S, et al Pulse pressure predicts cardiovascular risk in patients with type 2 diabetes mellitus. Am J Hypertens. 2005;18(11):1463–7; discussion 8–9. 10.1016/j.amjhyper.2005.05.009 . 16280282

[14] Anderson RJ, Bahn GD, Emanuele NV, Marks JB, Duckworth WC. Blood pressure and pulse pressure effects on renal outcomes in the Veterans Affairs Diabetes Trial (VADT). Diabetes Care. 2014;37(10):2782–8. Epub 2014/07/23. 10.2337/dc14-0284 25048382 PMC4170129

[15] Seow LSE, Subramaniam M, Abdin E, Vaingankar JA, Chong SA. Hypertension and its associated risks among Singapore elderly residential population. Journal of Clinical Gerontology & Geriatrics. 2015;6(4):125–32. 10.1016/j.jcgg.2015.05.002 WOS:000215862500004.

[16] Shi Y, Tham YC, Cheung N, Chua J, Tan G, Mitchell P, et al Is aspirin associated with diabetic retinopathy? The Singapore Epidemiology of Eye Disease (SEED) study. PLoS One. 2017;12(4):e0175966 Epub 2017/04/30. 10.1371/journal.pone.0175966 28453510 PMC5409055

[17] Lavanya R, Jeganathan VS, Zheng Y, Raju P, Cheung N, Tai ES, et al Methodology of the Singapore Indian Chinese Cohort (SICC) eye study: quantifying ethnic variations in the epidemiology of eye diseases in Asians. Ophthalmic Epidemiol. 2009;16(6):325–36. 10.3109/09286580903144738 . 19995197

[18] Foong AW, Saw SM, Loo JL, Shen S, Loon SC, Rosman M, et al Rationale and methodology for a population-based study of eye diseases in Malay people: The Singapore Malay eye study (SiMES). Ophthalmic Epidemiol. 2007;14(1):25–35. 10.1080/09286580600878844 . 17365815

[19] Tan GS, Gan A, Sabanayagam C, Tham YC, Neelam K, Mitchell P, et al Ethnic Differences in the Prevalence and Risk Factors of Diabetic Retinopathy: The Singapore Epidemiology of Eye Diseases Study. Ophthalmology. 2017 Epub 2017/12/09. 10.1016/j.ophtha.2017.10.026 . 29217148

[20] Grading diabetic retinopathy from stereoscopic color fundus photographs—an extension of the modified Airlie House classification. ETDRS report number 10. Early Treatment Diabetic Retinopathy Study Research Group. Ophthalmology. 1991;98(5 Suppl):786–806. Epub 1991/05/01. . 2062513

[21] Group ETDRSR. Grading diabetic retinopathy from stereoscopic color fundus photographs—an extension of the modified Airlie House classification. ETDRS report number 10. Early Treatment Diabetic Retinopathy Study Research Group. Ophthalmology. 1991;98(5 Suppl):786–806. . 2062513

[22] Sabanayagam C, Teo BW, Tai ES, Jafar TH, Wong TY. Ethnic differences in the association between blood pressure components and chronic kidney disease in middle aged and older Asian adults. BMC Nephrol. 2013;14:86 Epub 2013/04/18. 10.1186/1471-2369-14-86 PubMed Central PMCID: PMC3637167. 23590421 PMC3637167

[23] National Kidney F. K/DOQI clinical practice guidelines for chronic kidney disease: evaluation, classification, and stratification. Am J Kidney Dis. 2002;39(2 Suppl 1):S1–266. . 11904577

[24] Levey AS, Stevens LA, Schmid CH, Zhang YL, Castro AF, 3rd, Feldman HI, et al A new equation to estimate glomerular filtration rate. Ann Intern Med. 2009;150(9):604–12. 10.7326/0003-4819-150-9-200905050-00006 19414839 PMC2763564

[25] Tseng ST, Chou ST, Low BH, Su FL. Risk factors associated with diabetic retinopathy onset and progression in diabetes patients: a Taiwanese cohort study. Int J Clin Exp Med. 2015;8(11):21507–15. 26885099 PMC4723944

[26] Patel A, MacMahon S, Chalmers J, Neal B, Woodward M, Billot L, et al Effects of a fixed combination of perindopril and indapamide on macrovascular and microvascular outcomes in patients with type 2 diabetes mellitus (the ADVANCE trial): a randomised controlled trial. Lancet. 2007;370(9590):829–40. Epub 2007/09/04. 10.1016/S0140-6736(07)61303-8 . 17765963

[27] Estacio RO, Jeffers BW, Gifford N, Schrier RW. Effect of blood pressure control on diabetic microvascular complications in patients with hypertension and type 2 diabetes. Diabetes Care. 2000;23 Suppl 2:B54–64. . 10860192

[28] Schrier RW, Estacio RO, Jeffers B. Appropriate Blood Pressure Control in NIDDM (ABCD) Trial. Diabetologia. 1996;39(12):1646–54. 10.1007/s001250050629 . 8960857

[29] National Health Survey 2010, Singapore [Internet]. Available from: https://www.moh.gov.sg/docs/librariesprovider5/resources-statistics/reports/nhs2010—low-res.pdf.

[30] Huang OS, Tay WT, Ong PG, Sabanayagam C, Cheng CY, Tan GS, et al Prevalence and determinants of undiagnosed diabetic retinopathy and vision-threatening retinopathy in a multiethnic Asian cohort: the Singapore Epidemiology of Eye Diseases (SEED) study. Br J Ophthalmol. 2015;99(12):1614–21. Epub 2015/05/09. 10.1136/bjophthalmol-2014-306492 . 25953847

[31] Cui J, Ren JP, Chen DN, Xin Z, Yuan MX, Xu J, et al Prevalence and associated factors of diabetic retinopathy in Beijing, China: a cross-sectional study. BMJ Open. 2017;7(8):e015473 Epub 2017/09/01. 10.1136/bmjopen-2016-015473 . 28855199 PMC5724071

[32] Liu Y, Yang J, Tao L, Lv H, Jiang X, Zhang M, et al Risk factors of diabetic retinopathy and sight-threatening diabetic retinopathy: a cross-sectional study of 13 473 patients with type 2 diabetes mellitus in mainland China. BMJ Open. 2017;7(9):e016280 Epub 2017/09/03. 10.1136/bmjopen-2017-016280 28864696 PMC5588996

[33] Raman R, Gupta A, Krishna S, Kulothungan V, Sharma T. Prevalence and risk factors for diabetic microvascular complications in newly diagnosed type II diabetes mellitus. Sankara Nethralaya Diabetic Retinopathy Epidemiology and Molecular Genetic Study (SN-DREAMS, report 27). J Diabetes Complications. 2012;26(2):123–8. Epub 2012/03/27. 10.1016/j.jdiacomp.2012.02.001 . 22446033

[34] Zheng Y, Lamoureux EL, Lavanya R, Wu R, Ikram MK, Wang JJ, et al Prevalence and risk factors of diabetic retinopathy in migrant Indians in an urbanized society in Asia: the Singapore Indian eye study. Ophthalmology. 2012;119(10):2119–24. 10.1016/j.ophtha.2012.04.027 . 22709419

[35] Adler AI, Stratton IM, Neil HA, Yudkin JS, Matthews DR, Cull CA, et al Association of systolic blood pressure with macrovascular and microvascular complications of type 2 diabetes (UKPDS 36): prospective observational study. BMJ. 2000;321(7258):412–9. 10.1136/bmj.321.7258.412 10938049 PMC27455

[36] Longo-Mbenza B, Nkondi Mbadi ANJ, Mbungu Fuele S. Higher pulse pressure, systolic arterial hypertension, duration of diabetes and family history of diabetes in Central Africans. International Journal of Diabetes and Metabolism. 2008;16(1):17–23.

[37] Rema M, Pradeepa R. Diabetic retinopathy: an Indian perspective. Indian J Med Res. 2007;125(3):297–310. . 17496357

[38] Knudsen ST, Poulsen PL, Hansen KW, Ebbehoj E, Bek T, Mogensen CE. Pulse pressure and diurnal blood pressure variation: association with micro- and macrovascular complications in type 2 diabetes. Am J Hypertens. 2002;15(3):244–50. 10.1016/s0895-7061(01)02281-6 . 11939615

[39] de Simone G, Roman MJ, Alderman MH, Galderisi M, de Divitiis O, Devereux RB. Is high pulse pressure a marker of preclinical cardiovascular disease? Hypertension. 2005;45(4):575–9. Epub 2005/03/16. 10.1161/01.HYP.0000158268.95012.08 . 15767471

[40] Zhang G, Chen H, Chen W, Zhang M. Prevalence and risk factors for diabetic retinopathy in China: a multi-hospital-based cross-sectional study. Br J Ophthalmol. 2017 10.1136/bjophthalmol-2017-310316 . 28855195 PMC5754882

[41] Rajalakshmi R, Amutha A, Ranjani H, Ali MK, Unnikrishnan R, Anjana RM, et al Prevalence and risk factors for diabetic retinopathy in Asian Indians with young onset type 1 and type 2 diabetes. J Diabetes Complications. 2014;28(3):291–7. 10.1016/j.jdiacomp.2013.12.008 . 24512748

[42] Klein R, Klein BE, Moss SE, Davis MD, DeMets DL. Is blood pressure a predictor of the incidence or progression of diabetic retinopathy? Arch Intern Med. 1989;149(11):2427–32. Epub 1989/11/01. . 2684072

[43] Peralta CA, Shlipak MG, Wassel-Fyr C, Bosworth H, Hoffman B, Martins S, et al Association of antihypertensive therapy and diastolic hypotension in chronic kidney disease. Hypertension. 2007;50(3):474–80. Epub 2007/08/01. 10.1161/HYPERTENSIONAHA.107.088088 . 17664397

